# Cross‐wired metal stents for endoscopic bilateral stent‐in‐stent deployment in malignant hilar biliary obstruction: A multicenter, single‐arm, prospective study

**DOI:** 10.1002/deo2.20

**Published:** 2021-08-25

**Authors:** Kentaro Yamao, Takeshi Ogura, Hideyuki Shiomi, Takaaki Eguchi, Hisakazu Matsumoto, Zhao Liang Li, Hiroaki Hashimoto, Yasutaka Chiba, Mamoru Takenaka, Tomohiro Watanabe, Masatoshi Kudo, Tsuyoshi Sanuki

**Affiliations:** ^1^ Department of Gastroenterology and Hepatology Kindai University Hospital Osaka Japan; ^2^ The Second Department of Internal Medicine Osaka Medical College Osaka Japan; ^3^ Division of Gastroenterology, Department of Internal Medicine Kobe University Graduate School of Medicine Hyogo Japan; ^4^ Department of Gastroenterology and Hepatology Osaka Saiseikai Nakatsu Hospital Osaka Japan; ^5^ Department of Gastroenterology Japanese Red Cross Wakayama Medical Center Wakayama Japan; ^6^ Department of Gastroenterology Takarazuka City Hospital Hyogo Japan; ^7^ Department of Gastroenterology Bell Land General Hospital Osaka Japan; ^8^ Clinical Research Center Kindai University Hospital Osaka Japan; ^9^ Department of Gastroenterology Kita‐Harima Medical Center Hyogo Japan

**Keywords:** cholangiopancreatography, cholestasis, endoscopic retrograde, Klatskin tumor, self‐expandable metallic stents

## Abstract

**Objectives:**

The endoscopic bilateral stent‐in‐stent (SIS) deployment is a challenging procedure. Such difficulty is mainly caused by sticking of the tip of the delivery sheath into the self‐expandable metal stents (SEMSs) mesh, requiring an additional dilating procedure. Herein, we assessed the clinical results of using cross‐wired metal stent for endoscopic bilateral SIS deployment (BONASTENT M‐Hilar) in patients with malignant hilar biliary obstruction (MHBO) in both high‐volume and non‐high‐volume centers.

**Methods:**

We prospectively enrolled consecutive patients with MHBO between February 2016 and December 2018 at eight centers.

**Results:**

Forty‐six patients were enrolled during the study period. The proportions of technical success were 93.5% (43/46) and clinical success (CS) on intention‐to‐treat and per‐protocol analyses were 91.3% (42/46) and 93.0% (40/43), respectively. The proportion of an additional dilating procedure during the primary procedure was 50.0% (23/46). Recurrent biliary obstruction (RBO) on intention‐to‐treat analysis occurred in 32.6% (15/46) of cases. Almost all of the events were caused by stent ingrowth (14/15). The median survival time and time to RBO were 255 and 349 days, respectively. The probability of stent patency at 3, 6, and 12 months was 86.5%, 63.9%, and 47.6%, respectively.

**Conclusions:**

The cross‐wired metal stent had excellent technical and CS, although non‐high‐volume centers were included in this study (UMIN000021441).

## INTRODUCTION

Endoscopic biliary drainage is considered the best treatment option for patients with malignant hilar biliary obstruction (MHBO)^1^, ^2^, ^3^, ^4^, ^5^, ^6^ exhibiting jaundice; however, a couple of controversies exist regarding this procedure. The most controversial issue is the stenting methods utilizing self‐expandable metal stents (SEMSs). Regarding the stenting methods, the two stenting procedures widely used for bilateral biliary drainage in MHBO are side‐by‐side (SBS) and stent‐in‐stent (SIS). Both procedures have specific advantages and disadvantages, making the superiority of each debatable. A major disadvantage of SBS deployment is overexpansion of the biliary stricture and distal bile duct by insertion of two SEMSs.^7^ Similarly, a major disadvantage of SIS deployment is utilizing through the mesh (TTM) technique with guidewires, requiring highly experienced personnel for placing the second SEMS delivery sheath into the first during the primary procedure. Expectedly, reintervention for a primary SEMS obstruction with the TTM technique is considered very difficult, especially when additional SEMS insertion into the primary first SEMS is required. However, previous reports showed a high proportion of technical success (TS, 86.7%–100%) for primary SIS deployment in MHBO.^8^, ^9^, ^10^, ^11^, ^12^, ^13^, ^14^, ^15^ Furthermore, in a recent reports, the proportion of TS for reintervention was also high (85.2%–92.3%) in cases exhibiting primary SEMS obstruction.^14^, ^16^ This high TS rate can be partially explained by the recent development of SEMSs dedicated for SIS deployment using the TTM technique. However, these high TS rates need to be interpreted with caution, as these results were obtained in high‐volume centers. In this multicenter study, we assessed the clinical results of using a cross‐wired metallic stent for endoscopic bilateral SIS deployment in patients with MHBO including non‐high‐volume centers.

## MATERIALS AND METHODS

### Patients

Patients with the following inclusion criteria were enrolled between February 2016 and December 2018: (1) pathologically diagnosed unresectable MHBO with Bismuth classification type II, III, and IV^17^; (2) age > 20 years; and (3) Eastern Cooperative Oncology Group performance status 0–3.^18^ The exclusion criteria were (1) history of biliary surgery (2) severe dysfunction in other organs (American Society of Anesthesiologist's physical status grade III or IV^19^); (3) life expectancy ≤ 3 months; (4) severe cancer spread with insufficient margin at the intrahepatic and/or papillary side; (5) judged to be ineligible by the investigator; or (6) declining to participate in the study. The Review Boards of all eight participating centers approved the study, which was performed according to the guidelines of the Helsinki Declaration for Biomedical Research Involving Human Subjects (Clinical trial registration number: UMIN000021441). All patients provided written informed consent.

### Equipment and procedure

**FIGURE 1:**
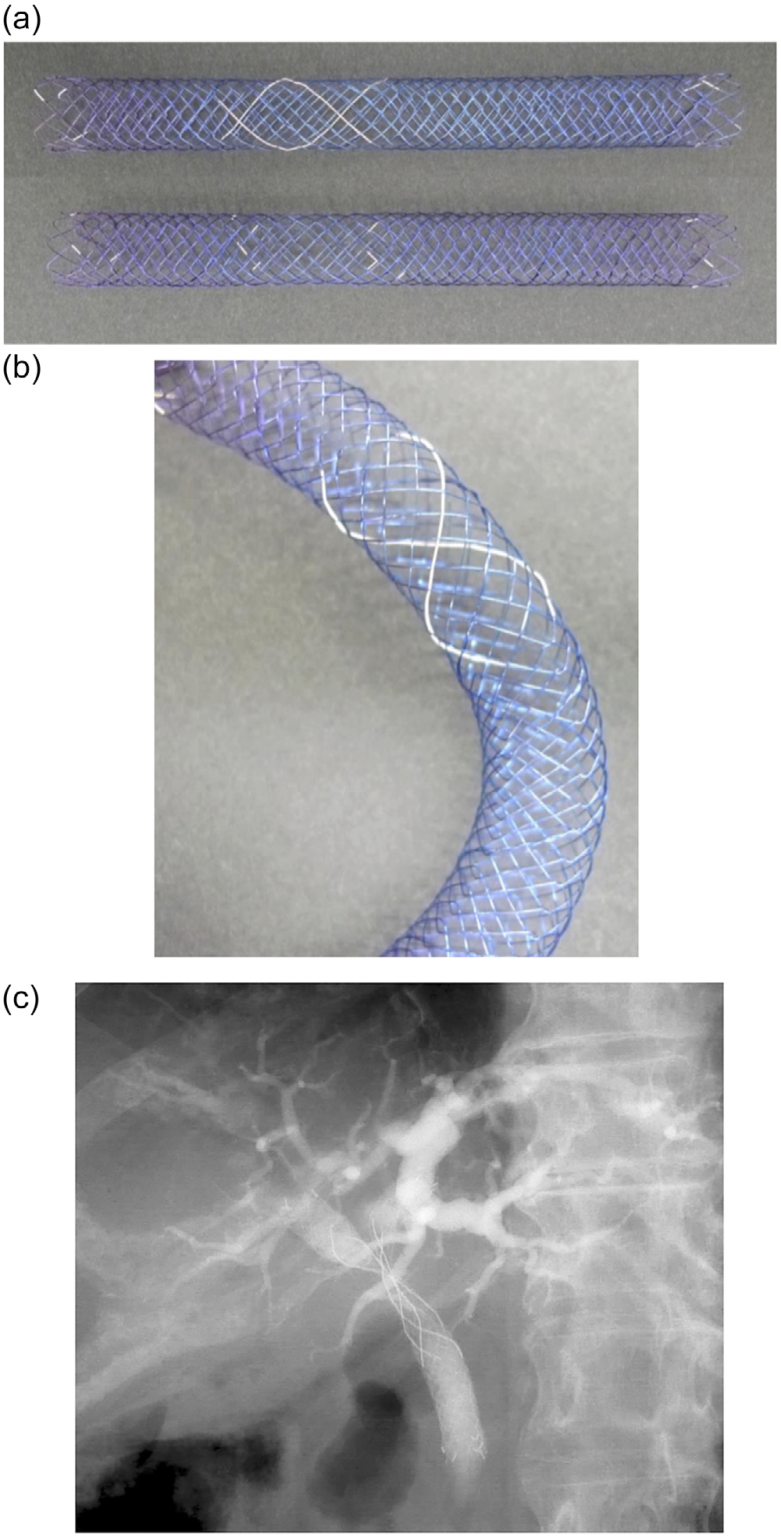
(a and b) The appearance of the cross‐wired metal stent for stent‐in‐stent deployment, and (c) representative bile duct image after deployment of self‐expandable metal stent deployment

We used the Cross‐wired Metallic Stent for Endoscopic Bilateral SIS (BONASTENT M‐Hilar; 8 and 10 mm diameter, length of 40, 50, 60, 70, 80, and 100 mm with 15‐, 20‐, or 25‐mm long central part; Standard Sci‐Tech, Seoul, South Korea). The woven hook and cross‐wired structure of this SEMS act together to produce high radial force and low axial force on the proximal and distal portions (Figure 1a). Additionally, its central part comprises a cross‐wired structure suitable for insertion of the second SEMS. The central part has radiopaque markers enabling the first SEMS to identify the origin of the contralateral bile duct easily (Figure 1b).

A drainage area was determined based on the assessment of liver volume and morphology of bile duct using computed tomography and magnetic resonance cholangiopancreatography imaging. All endoscopic procedures were performed under conscious sedation with midazolam or propofol. A duodenal endoscope (TJF‐260V, JF‐260V; Olympus Optical, Tokyo, Japan) and an endoscopic retrograde cholangiopancreatography catheter (Tandem XL; Boston Scientific, Massachusetts, USA, or MTW; MTW Endoskopie, Düsseldorf, Germany) were used. After successful biliary cannulation, manipulation of the targeted bilateral intrahepatic branches across the hilar stricture was performed using a 0.025‐inch wire (VisiGlide; Olympus Optical, VisiGlide 2; Olympus Optical, M‐through; Asahi Intecc, Aichi, Japan, Radifocus; Terumo, Tokyo, Japan) or a 0.035‐inch wire (Jagwire; Boston Scientific, Revowave; Piolax Medical Devices, Kanagawa, Japan). A contrast medium was injected into the bile duct to identify obstruction sites. Balloon dilation (REN biliary dilation catheter; KANEKA, Osaka, Japan, ZARA; Century Medical, Tokyo, Japan) and catheter dilation (Soehendra Biliary Dilation Catheter, Cook Medical, North Carolina, USA) were performed in case of severe stenosis. The first SEMS was deployed to cover the stenosis, and the cross‐wired central part was positioned at the origin of the contralateral bile duct. Then, the guidewire was inserted into the contralateral side with the TTM of the first SEMS followed by deployment of the second SEMS in an SIS procedure. If guidewire manipulation with the TTM was difficult, balloon dilation of the first SEMS was repeatedly performed to expand the first SEMS mesh. The number of deployed SEMS was two; insertion of the third SEMS was not attempted.

### Outcome measurements and definitions

Our primary objective was to assess the proportion of TS. Clinical success (CS), recurrent biliary obstruction (RBO), adverse events (AE), additional dilating procedure during SEMS deployment, survival time and time to RBO (TRBO), and the probability of RBO at 3, 6, and 12 months were included as secondary objectives. Clinical results of patients with RBO who required reintervention were also assessed. TS was defined as adequate SEMS deployment with an SIS method into two bile ducts. CS was defined as normalized levels or reduced levels (≥50%) of serum bilirubin within 2 weeks. Survival time was measured from the day of SEMS deployment to death. All patients were followed up until death or the end of the study period, but patients who were lost to follow‐up evaluation were censored for survival time. TRBO was measured as the day of SEMS deployment to RBO. Patients who died or met follow‐up evaluation without RBO occurrence were censored for TRBO measurement. RBO and AE were defined according to the TOKYO criteria for trans‐papillary biliary stenting.^20^ Early and late AEs were defined as stent or procedure‐related AEs within or after 30 days of SEMS placement. AE severity was graded according to the American Society of Gastrointestinal Endoscopy lexicon.^21^ An additional dilating procedure during SEMS deployment was noted when there was a requirement for performing the balloon and/or catheter dilation when the first SEMS device was not smoothly passed in the bile duct stenosis or TTM of the second SEMS in the first SEMS mesh. The procedure time was defined as the minutes between the insertion and removal of the endoscope. High‐volume and non‐high‐volume centers were defined as a hospital with more than and fewer than annual 700 ERCP procedures in the study period, respectively.

### Sample size calculation

Kogure et al^11^ reported that the proportion of TS in SIS deployment was 96%. Therefore, we hypothesized that the threshold proportion of TS was 80%, and the expected probability was 90% because high‐volume and non‐high‐volume centers participated in this study. The necessary number of patients was set to be 55 with a significance level of 0.05 and a power of 0.8 based on these assumption.

### Statistical analyses

Intention to treat (ITT) and per‐protocol (PP) analysis methods were used in this study. The ITT analysis was performed based on the original total cohort of enrolled patients. The baseline characteristics and the proportion of TS, early AE and survival time were evaluated by ITT analysis. The PP analysis was performed based on the subset of patients in whom bilateral stenting was successful. The proportions of CS, RBO, TRBO, and late AE were evaluated by both ITT and PP analyses.

Mean and standard deviation were used to describe continuous variables, while percentages were used for categorical variables. The probability of patient survival and RBO were estimated using the Kaplan‐Meier method. Statistical analyses were performed using GraphPad Prism 8 (GraphPad Software Inc., San Diego, CA, USA).

## RESULTS

**FIGURE 2:**
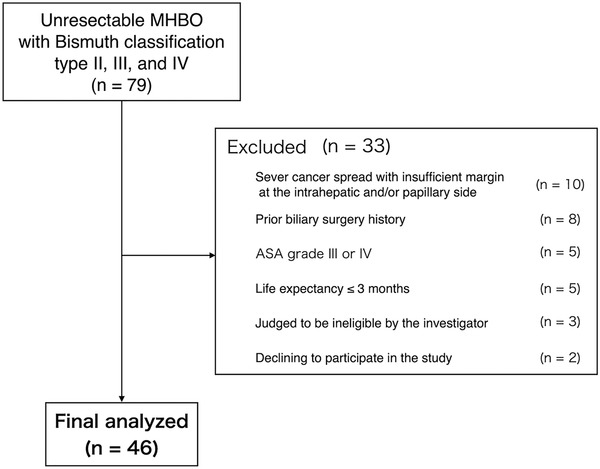
Flow chart of the selection of patients in the study

Patient recruitment was performed from February 2016 to December 2018 at eight hospitals: four high‐volume centers (Kindai University Hospital, Osaka Medical College Hospital, Kobe University Hospital and Japanese Red Cross Wakayama Medical Center) and four non‐high‐volume centers (Osaka Saiseikai Nakatsu Hospital, Takarazuka City Hospital, Bell Land General Hospital and Kita‐Harima Medical Center). The final follow‐up was completed in June 2019 Although the number of required patients per sample size calculation was 55, we enrolled and assessed 46 patients who were eligible during the study period (Figure 2).

### Patients’ characteristics

Tumor diagnoses were cholangiocarcinoma in 26 patients (56.5%), gallbladder cancer in nine (19.6%), and others in 11 (23.9%). Although the tumor stage was nonresectable stage in most patients (93.5%), some of the resectable‐stage patients received endoscopic drainage alone because of advanced age or underlying diseases. The number of patients in the high‐volume and non‐high‐volume centers was 37 (80.4%) and nine (19.6%), respectively (Table 1).

**TABLE 1:** Patients’ demographic and clinical characteristics (*n* = 46)

Age, median (range), years	77.5 (56–92)
Sex, *n* (%)	
Male	24 (52.2)
Female	22 (47.8)
Tumor etiology, *n* (%)	
Cholangiocarcinoma	26 (56.5)
Gallbladder cancer	9 (19.6)
Lymph node metastasis from a cancer at another site	5 (10.9)
Pancreatic cancer	3 (6.5)
Others	3 (6.5)
Tumor stage, *n* (%)	
Resectable	3 (6.5)
Locally advanced	10 (21.7)
Metastatic	33 (71.7)
Performance status, *n* (%)	
0‐2	45 (97.8)
3	1 (2.2)
Bismuth classification, *n* (%)	
II	17 (37.0)
IIIa	2 (4.3)
IIIb	5 (10.9)
IV	22 (47.8)
Previous biliary drainage, *n* (%)	
None	13 (28.3)
Plastic stent	16 (34.8)
ENBD*	8 (17.4)
Plastic stent + ENBD*	9 (19.6)
Chemotherapy after stenting, *n* (%)	15 (32.6)
Hospital, n (%)	
High‐volume care referral center	37 (80.4)
Non‐high‐volume center	9 (19.6)

Abbreviation: ENBD, endoscopic nasobiliary drainage.

### TS and CS of SEMS deployment

The proportion of TS in a single session in all the patients was 93.5%, and all cases with TS were two deployed SEMSs. The proportions of TS in the high‐volume and non‐high‐volume centers were 91.9% and 100%, respectively. Three patients experienced technical failure despite additional balloon dilatation due to difficulty in passing the first SEMS delivery sheath into duct stenosis, the TTM of the guidewire into the first SEMS, and the TTM of the second SEMS delivery sheath. Reinsertion of SEMS in the second session was not attempted in cases with difficulty in primary procedures. Regarding the clinical courses of the patients with technical failure, one patient was treated with deployment of single plastic stent (PS) without the achievement of CS. One patient achieved CS by deployment of PS with SIS deployment as the second stent and the other patient achieved CS without the deployment of the second stent (single SEMS only). An additional dilating procedure during SEMS deployment was required in 23 patients (50.0%); dilatation of bile duct stenosis before first SEMS deployment was required in five (10.9%); and dilation of the first SEMS and/or SEMS mesh before the second SEMS deployment was required in 19 patients (41.3%), with overlapping in one patient. CS was achieved in 42 patients including all three patients with technical failure on ITT analysis (91.3%) and 40 in 43 patients with TS on PP analysis (93.0%) (Table 2).

**TABLE 2:** Overall outcomes of endoscopic bilateral stent‐in‐stent placement (*n* = 46)

Technical success, *n* (%)	43 (93.5)
Technical success in high‐volume care referral center	34 (91.9)
Technical success in non‐high‐volume center	9 (100)
Technical failure, *n* (%)	3 (6.5)
Failure in the passage of first SEMS delivery into bile duct stenosis	1
Failure in guidewire insertion into first SEMS mesh	1
Failure in the passage of second SEMS delivery into first SEMS mesh	1
Procedure time, median (range), minutes*	48 (15–131)
Additional dilation procedure, *n* (%)	23 (50.0)
Dilation of bile duct stenosis before first SEMS deployment	5 (10.9)
Dilation of first SEMS and/or SEMS mesh before second SEMS deployment	19 (41.3)
Clinical success on ITT analysis, *n* (%)	42 (91.3)
Clinical success on PP analysis, *n* (%)	40/43 (93.0)

Abbreviations: ITT, intention‐to‐treat; PP, per‐protocol; SEMS, self‐expandable metal stent.

*Including the patients with technical failure.

### Proportions of RBO and AE

RBO on ITT analysis occurred in 15 patients (32.6%) due to stent ingrowth (14 patients) and stent overgrowth (one patient). All stent ingrowth occurred in the central part of the SEMS. Reintervention was required for all 15 patients. Early AE on ITT analysis occurred in two patients (4.3%) due to post‐ERCP pancreatitis. Neither late AEs nor procedure‐related mortality was observed (Table 3).

**TABLE 3:** Recurrent biliary obstructions and adverse events (*n* = 46)

RBO on ITT analysis, *n* (%)	15 (32.6)
Causes of RBO	
Stent ingrowth, *n* (%)	14 (30.4)
Stent overgrowth, *n* (%)	1 (2.2)
RBO on PP analysis, *n* (%)	14/43 (32.6)
Causes of RBO	
Stent ingrowth, *n* (%)	13/43 (30.2)
Stent overgrowth, *n* (%)	1/43 (2.3)
Early adverse events on ITT analysis, *n* (%)	2 (4.3)
Pancreatitis, *n* (%)	2 (4.3)
Late adverse events on ITT analysis, *n* (%)	0 (0)
Late adverse events on PP analysis, *n* (%)	0/43 (0)

Abbreviations: ITT, intention‐to‐treat; PP, per‐protocol; RBO, recurrent biliary obstruction.

### Probability of patient survival and RBO

**FIGURE 3:**
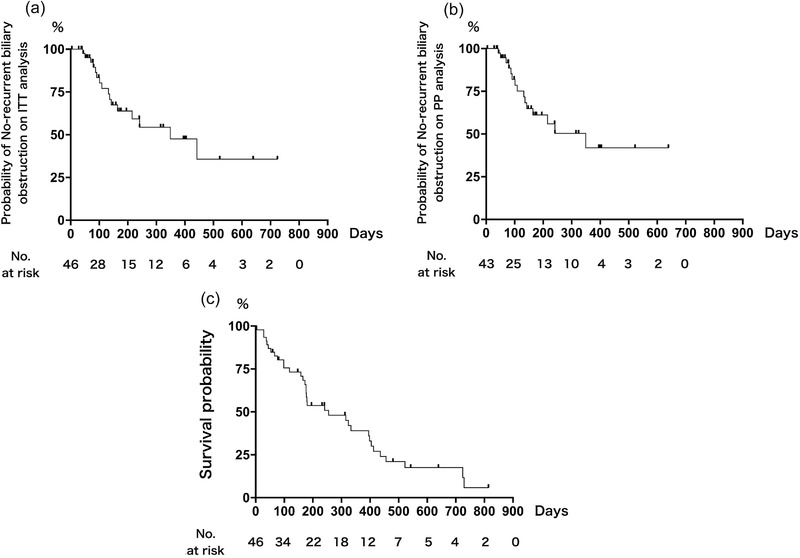
Kaplan‐Meier curves for (a) time to recurrent biliary obstruction on intention‐to‐treat analysis, (b) per‐protocol analysis, and (c) patient survival

Patient survival was monitored until May 2019. Five patients were lost to follow‐up evaluation, and seven were alive at the end of the observation period. The median survival time was 255 days and TRBO 349 days on both ITT and PP analyses, respectively. The probabilities of stent patency at 3, 6, and 12 months on ITT and PP analyses were 86.5%, 63.9%, and 47.6%, as well as 85.3%, 61.1%, and 42.0% on PP analysis, respectively (Figures 3a, b, and c).

### Clinical results of reintervention

Reintervention was required in 15 patients (32.6%). Although TS, defined as stent deployment, was achieved in all 15 patients (100%), the deployment as a reintervention plan was seen in 12 patients (80.0%). The reason for technical failure, reintervention planned failure, was difficulty in inserting the catheter or SEMS delivery into the first SEMS of the primary procedure, despite SEMS mesh dilation. Both sides were deployed in six patients (SEMS in two and PS in four), and one side deployment was seen in nine patients (SEMS in three and PS in six). Additional dilating procedures were required in nine patients (60.0%).

## DISCUSSION

This multicenter prospective study assessed the clinical results using a cross‐wired metallic stent for endoscopic bilateral SIS deployment in patients with MHBO. Although SIS deployment is a challenging endoscopic procedure, we expected a high proportion of TS due to the presence of the central cross‐wired part, which enabled easy insertion of the second SEMS into the first. Although non‐high‐volume centers were included in this study, the proportion of TS and CS was high in this study. However, we have to be cautious regarding the interpretation of these data since we could not enroll sufficient number of patients adequate for appropriate and statistical assessment.

The method of endoscopic procedure in MHBO is controversial. Among the typical methods for multiple stenting, the suitability of the widely used SIS or SBS deployment in patients with MHBO is debatable. One advantage of SIS technique is achieving multiple SEMS placement in one stent caliber at the common bile duct, making it physiologically ideal in terms of the bile flow. However, the TTM technique with guidewires followed by the SEMS delivery sheath can sometimes be technically difficult in SIS. Alternatively, the SBS technique is simple at both initial placement and reintervention upon stent occlusion. However, potential disadvantages of SBS include overexpansion of the biliary stricture and/or frequent portal vein thrombosis because of parallel placement of multiple SEMSs.^7^ In a previous report on SIS deployment, the proportions of TS in a single session and in the final session ranged from 77.1% to 100% and 86.7% to 100%, respectively.^8^, ^9^, ^10^, ^11^, ^12^, ^13^, ^14^, ^15^ Furthermore, an additional balloon dilating procedure of the TTM during the second SEMS deployment into the first SEMS was required in 22.5%^10^ and 23.1% of patients^11^, respectively. This additional balloon dilating process might be avoided by using a moving cell stent as previously reported.^13^ Thus, these previous studies highlight the advantages rather than disadvantages of SIS for patients with MHBO, although the SIS procedure is technically more difficult than the SBS procedure. Similarly, we have provided evidence that patients with MHBO are successfully treated with endoscopic bilateral SIS deployment using a cross‐wired metallic stent. One major concern of this novel procedure is the high percentage (50.0%) of patients requiring balloon and/or catheter dilation during SEMS deployment despite the presence of the cross‐wired central part. However, the second SEMS in SIS deployment was successfully placed in almost all patients after the additional dilating procedure.

During reintervention for RBO, the insertion of SEMS or PS and TTM technique are frequently challenging. Regarding reintervention for RBO after bilateral drainage in MHBO, the proportions of TS and CS were reported to be 92% and 90%, respectively.^22^ In our study, the proportion of TS of stent deployment was 100%, and almost all patients underwent successful stent deployment as a reintervention plan, although an additional dilating procedure was required. Although it was difficult to perform the TTM technique into the first SEMS of the primary procedure using a conventional stent, the presence of the cross‐wired central part enabled successful stent deployment in patients requiring reintervention.

Stent ingrowth was the primary factor for RBO in this study. All cases with stent ingrowth were seen in the central part of the SEMS, probably because the SEMS used for SIS deployment is the uncovered one, and the central part has a sparse structure to facilitate the TTM technique. In a previous report on SIS deployment, the proportions of overall stent dysfunction (SD) and stent ingrowth were 30.8%–63.2%^9^, ^10^, ^11^, ^12^, ^14^, ^15^, ^23^ and 5.0%– 44.7%^11^, ^12^, ^15^, ^23^, respectively. In this study, the proportion of RBO was 32.6%, and most of the occluded SEMS‐events were caused by stent ingrowth all in the central cross‐wired part. SBS deployment using fully covered SEMS with a small diameter (6 mm)^16^ and inside PS^24^, ^25^, ^26^, ^27^ was recommended in previous reports to prevent stent ingrowth. Indeed, no stent ingrowth occurred using these stents despite SD by sludge.^16^, ^24^, ^25^, ^26^ Additionally, Kanno et al have reported that the TRBO was significantly longer in the inside PS group than that in the SEMS group.^27^ Thus, these previous studies provide evidence that placement of PS is superior to that of SEMS for the prevention of stent ingrowth. However, it is too early to determine the superiority of PS placement over SEMS placement to prevent SD since there is no prospective study comparing uncovered SEMS for SIS, uncovered or fully covered SEMS, and inside PS for SBS.

We used the same cross‐wired metal stent used in previous reports.^8^, ^10^, ^14^, ^15^ The differences between the present and previous studies were the participating facilities; the previous study was conducted at academic high‐volume centers,^8^, ^10^, ^14^, ^15^ whereas this study was conducted in both high‐volume and non‐high‐volume centers. In previous studies, the proportions of using the cross‐wired metal stent in a single session and the final session were 77.1%–100%^8^, ^10^, ^15^ and 78.6%–100%^8^, ^10^, ^14^, ^15^, respectively. The proportion of TS in our study was 93.5%, and all patients with TS underwent a single session. Although non‐high‐volume centers participated in this study, we achieved a high proportion of TS.

Our study had some limitations. First, the number of enrolled patients did not meet the target sample size. Second, the study had a single‐arm design. Finally, 50% of the patients required an additional dilating procedure, but the methods differed.

In conclusion, endoscopic bilateral SIS deployment using the cross‐wired metal stent has achieved excellent TS when combined with an additional dilation procedure in patients with MHBO. The data obtained in this study, including both high‐volume and non‐high‐volume centers, provide real world data to assess the utility and safety of endoscopic bilateral SIS deployment using the cross‐wired metal stent in patients with MHBO.

## CONFLICT OF INTEREST

The authors declare that they have no conflict of interest.

## FUNDING INFORMATION

None.
